# Pronation-Type Galeazzi-Equivalent Fracture: A Case Report

**DOI:** 10.7759/cureus.43430

**Published:** 2023-08-13

**Authors:** Ali M Alahmari, Haneen Hafiz, Abdulrhman M Alfaidi, Ahmed Ali

**Affiliations:** 1 Orthopedics, King Fahad General Hospital, Jeddah, SAU; 2 Orthopedics, Security Forces Hospital, Makkah, SAU; 3 Orthopedic Surgery, Prince Meshari Bin Saud-General Hospital, Baljurashi, SAU; 4 Orthopedic Surgery, King Abdullah Medical Complex, Jeddah, SAU

**Keywords:** pronation type, distal forearm pediatric fracture, galeazzi fracture, galeazzi equivalent, distal radiounlar joint

## Abstract

Fractures of the forearm are common among children and adolescents. Radial shaft fracture with dislocation of the distal radioulnar joint (DRUJ), called Galeazzi fracture, is unusual in pediatrics. The Galeazzi-equivalent fracture is a variant of the classic Galeazzi fracture that occurs in children and adolescents. It is a radius fracture associated with a distal ulnar displaced physeal injury without dislocation of the DRUJ. Our patient was a male, aged 15 years, who visited our emergency department after falling off a scooter onto his left hand. Left wrist X-rays showed a displaced Galeazzi-equivalent fracture. After a trial of close reduction, an X-ray showed a displaced and unstable fracture pattern. The patient was subsequently hospitalized for surgical intervention. Open reduction and internal fixation (ORIF) with a plate and screw were used for the radius fracture. The ulna fracture was irreducible; therefore, ORIF with two crossed smooth Kirschner wires (K-wires) was performed. Complete bone union was achieved, and he had a normal range of motion six months postoperatively. The patient is now able to perform daily and sports activities. At two-year follow-up, complications such as DRUJ instability or joint deformity did not occur. In conclusion, open reduction is desired for patients with malalignment or older patients who have a lower potential for sufficient bone remodeling. Regular serial follow-up sessions are required to assess growth arrest and the occurrence of other complications.

## Introduction

Fractures of the forearm are common among children and adolescents. Radial shaft fracture with dislocation of the distal radioulnar joint (DRUJ), called Galeazzi fracture, is unusual in pediatric cases and was reported to occur in <3% of all forearm pediatric fractures [1], especially in 9- to 13-year-olds [2]. There is a variation of this lesion: radial fracture associated with distal ulnar displaced physeal injury without dislocation of DRUJ, which is stabilized using a distal ligamentous stabilizing system (DLSS), including distal volar and dorsal radioulnar ligaments and triangular fibrocartilage complex. In immature bones, the physis is weaker and less resistant than the DLSS [3]. This type is called Galeazzi-equivalent fracture.

The classification of pediatric Galeazzi injuries was described by Walsh and McLaren based on the direction of the displacement of the distal radial fracture. A more typical injury pattern is due to supination force, where the dorsal displacement of the distal radius occurs, in which the distal ulna lies volar. The least frequent pattern is due to pronation force, which includes volar displacement of the distal radius and the distal ulna lying dorsally [2]. Our case was a pronation type, with a distal radius fracture with apex dorsal angulation, associated with a volar displacement of the ulnar epiphysis due to intact DLSS, and the ulnar metaphyseal displaced dorsally.

Galeazzi-equivalent fractures are not difficult to recognize but can be missed at first presentation. Proper wrist images and wrist examination are mandatory to diagnose DRUJ injury and distal ulnar physeal injury [2-4]. Complications of the Galeazzi-equivalent fracture include malunion, nonunion, chronic DRUJ instability, and ulnar growth arrest [5].

## Case presentation

**Figure 1:**
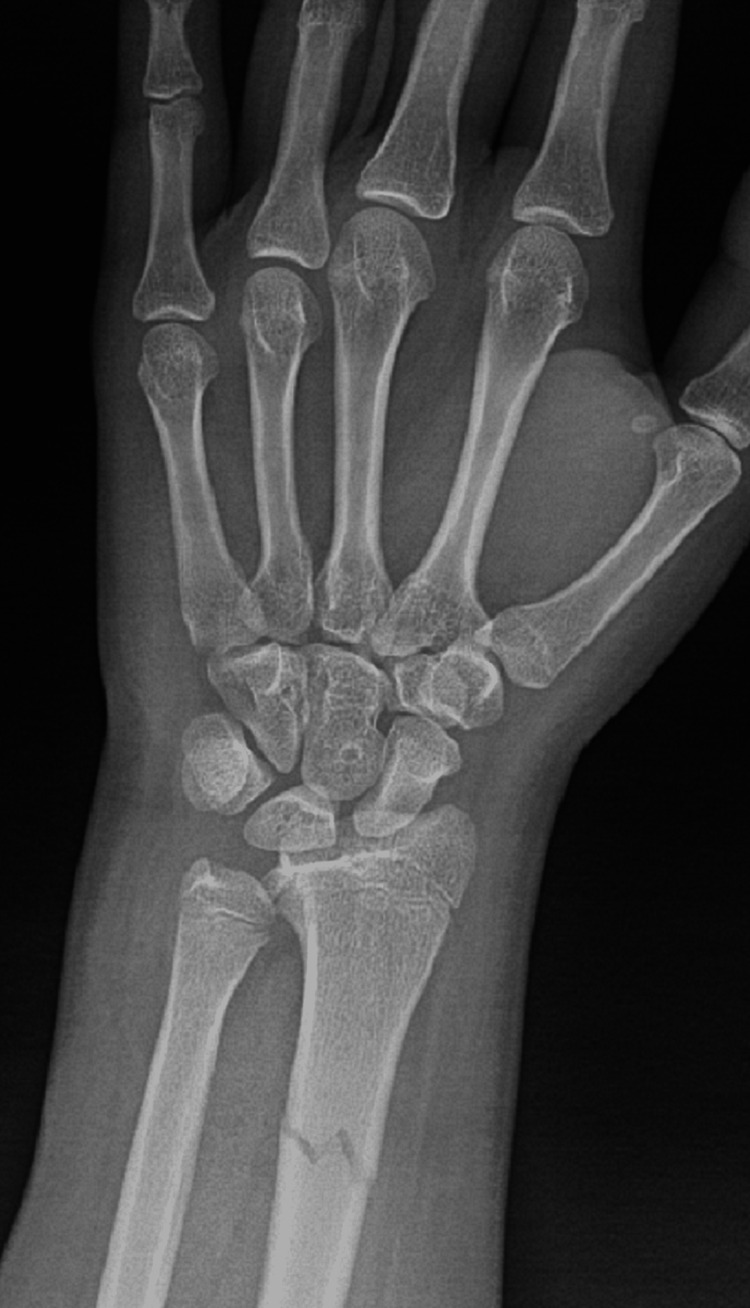
PA wrist view of Galeazzi-equivalent fracture. PA: posterior-anterior.

**Figure 2:**
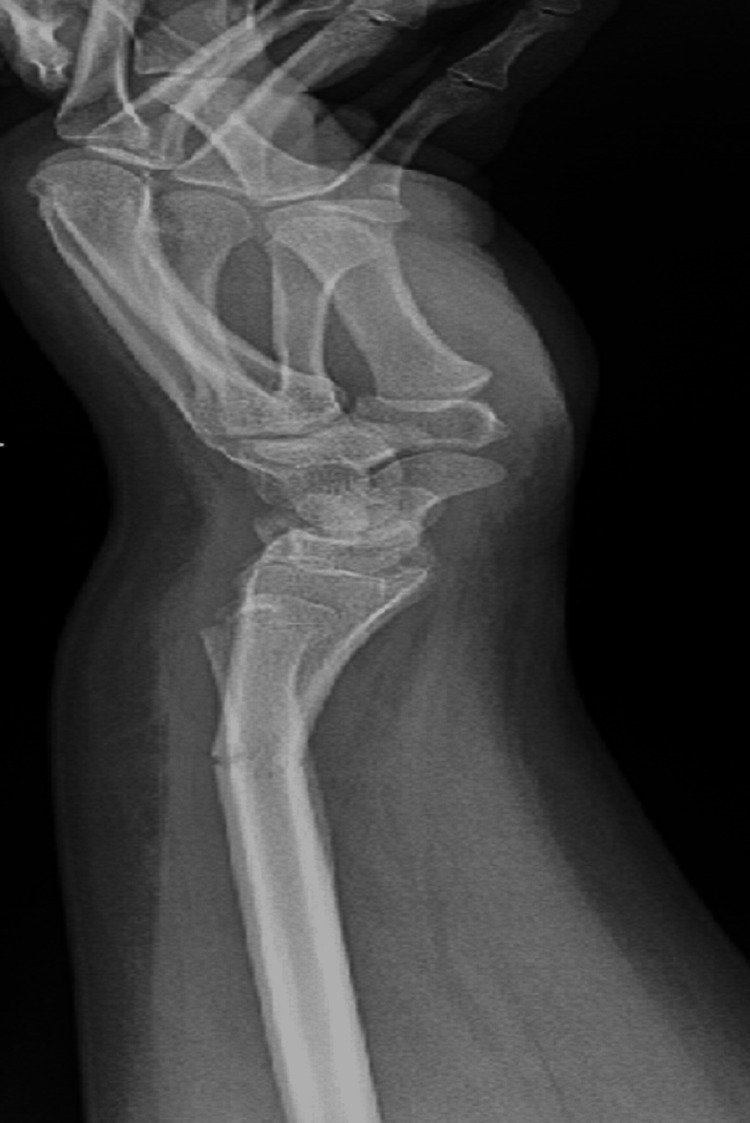
Lateral wrist view of Galeazzi-equivalent fracture.

A right-handed 15-year-old male with no known illnesses or surgical history fell off his scooter onto his left hand. He came to our emergency department complaining of left wrist pain and swelling as an isolated complaint. He had no history of old trauma. A left wrist examination showed mild swelling, no wounds, tenderness over the left distal forearm, and a limited range of motion of the left wrist, and a distal neurovascular examination was intact. A left wrist X-ray, posterior-anterior (PA) view, showed a transverse nondisplaced distal third radius shaft fracture (Figure 1). The lateral wrist view showed a distal third radius fracture with dorsally apex angulation of 21°, and distal ulna fracture with minimally displaced volar around 2.5 mm, Salter-Harris classification type II (Figure 2).

**Figure 3:**
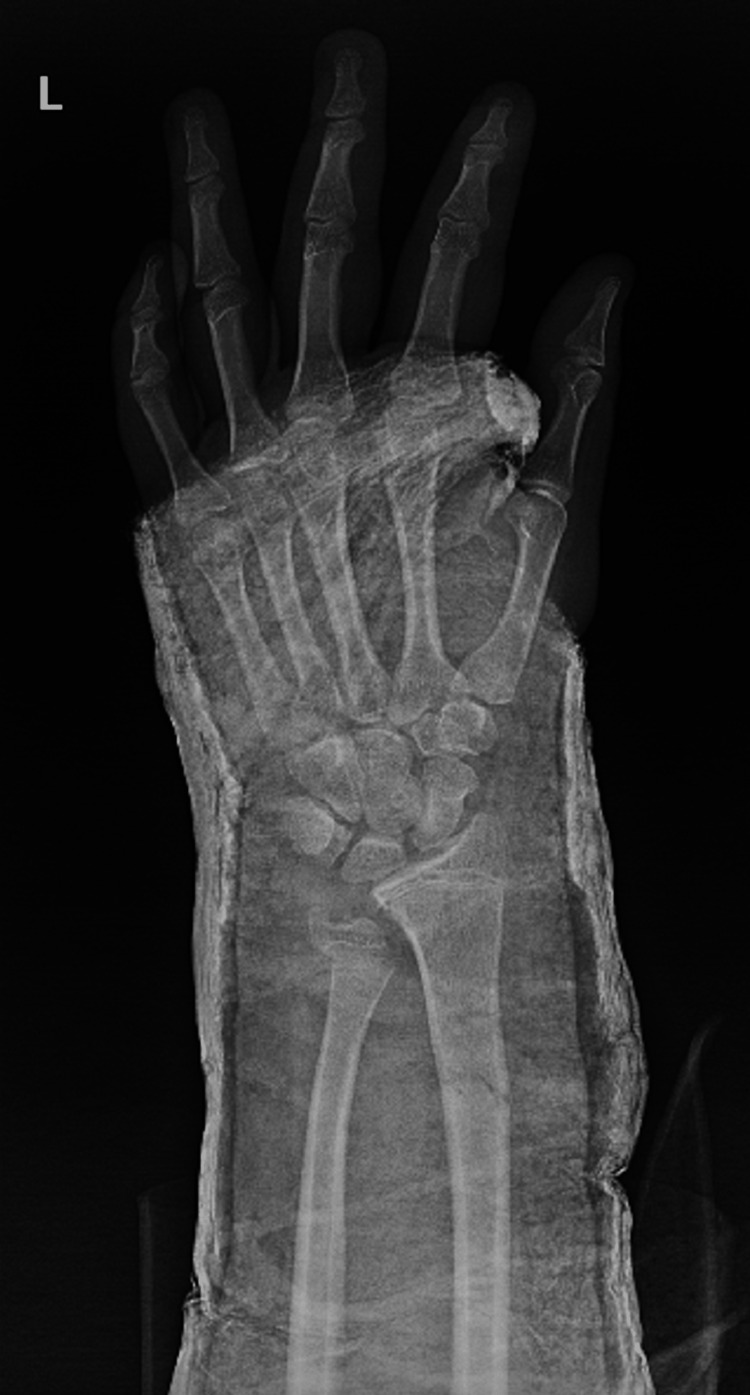
PA wrist view after a trial of reduction. PA: posterior-anterior.

**Figure 4:**
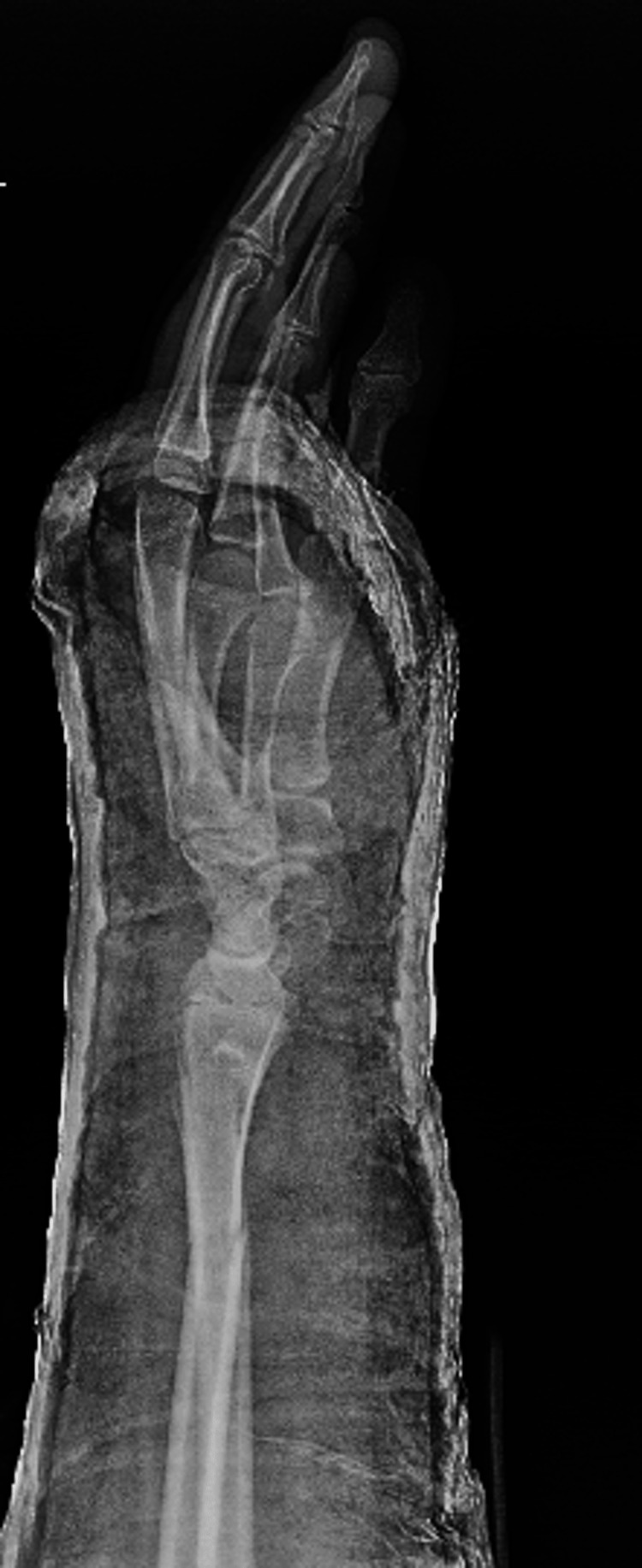
Lateral wrist view after a trial of reduction.

**Figure 5:**
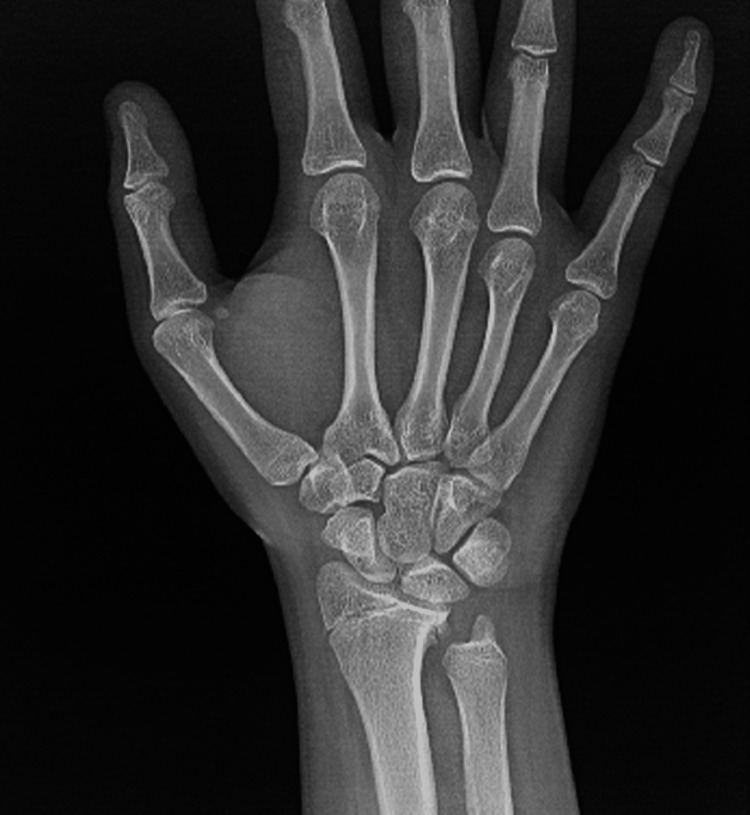
PA view of the right wrist. PA: posterior-anterior.

**Figure 6:**
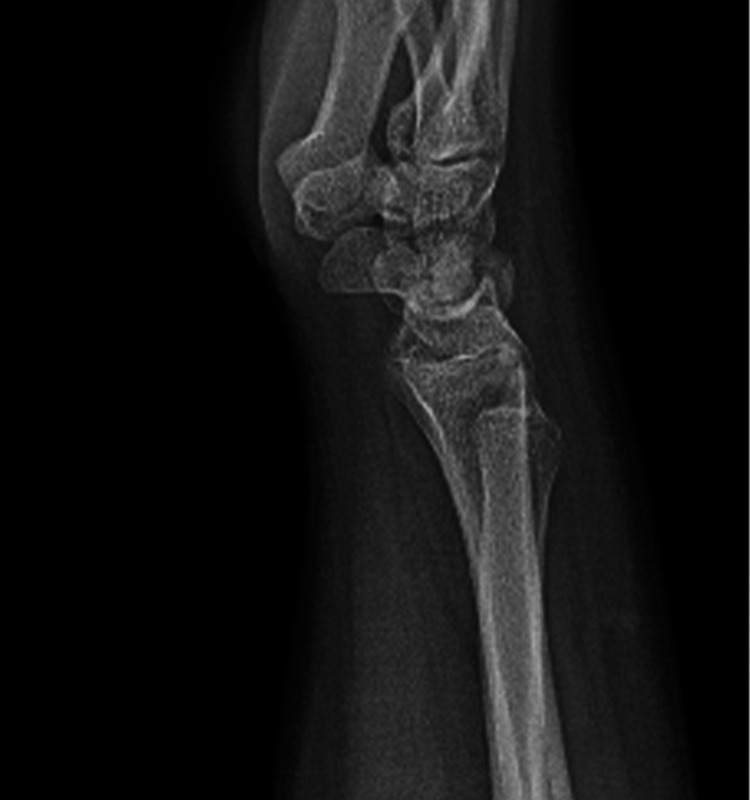
Lateral view of the right wrist.

After a trial of close reduction, true PA and lateral left wrist X-rays showed a displaced and unstable fracture pattern with a negative ulnar variance of 4 mm (Figures 3, 4). The right wrist X-ray showed a negative ulnar variance of around 5 mm, comparable to the affected side (Figures 5, 6).

**Figure 7:**
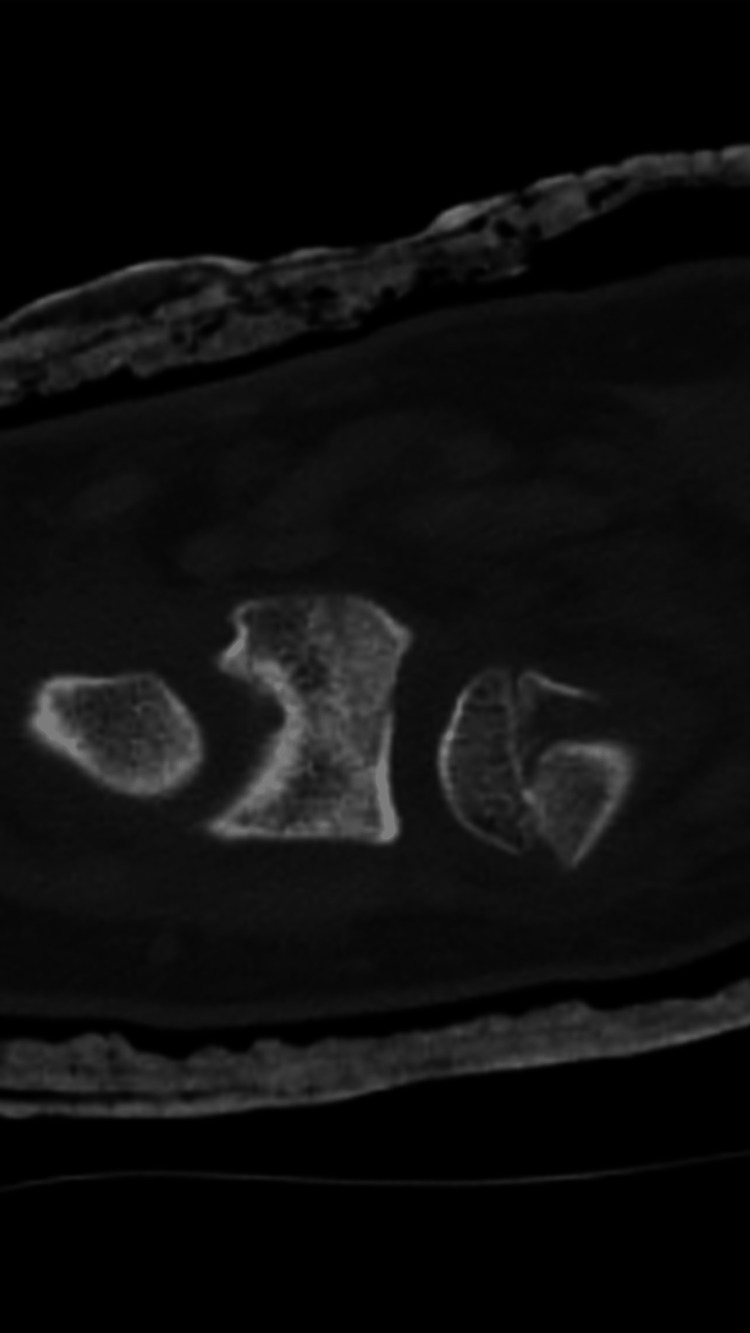
CT imaging of the left wrist in the sagittal plane. CT: computed tomography.

**Figure 8:**
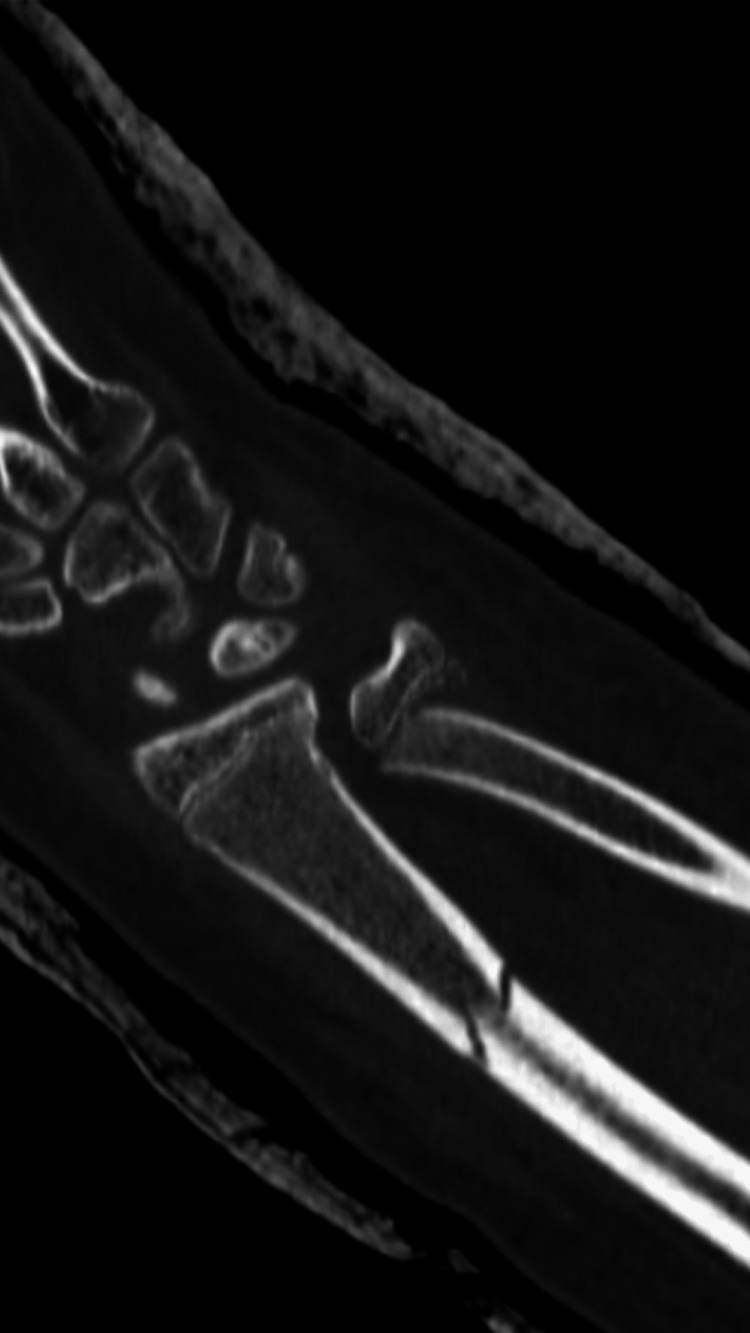
CT imaging of the left wrist in the coronal plane. CT: computed tomography.

A computed tomography (CT) scan of the left wrist showed a distal ulna fracture, Salter-Harris classification type II, with a displaced volar of 2.5 mm in the sagittal plane and displaced by 3.5 mm in the coronal plane (Figures 7, 8).

The patient was admitted to the hospital and all preoperative investigations were done. The patient was examined by the anesthesia team, and he was ready for the operation. We explained expected complications such as distal ulna physeal arrest and deformity to his family.

**Figure 9:**
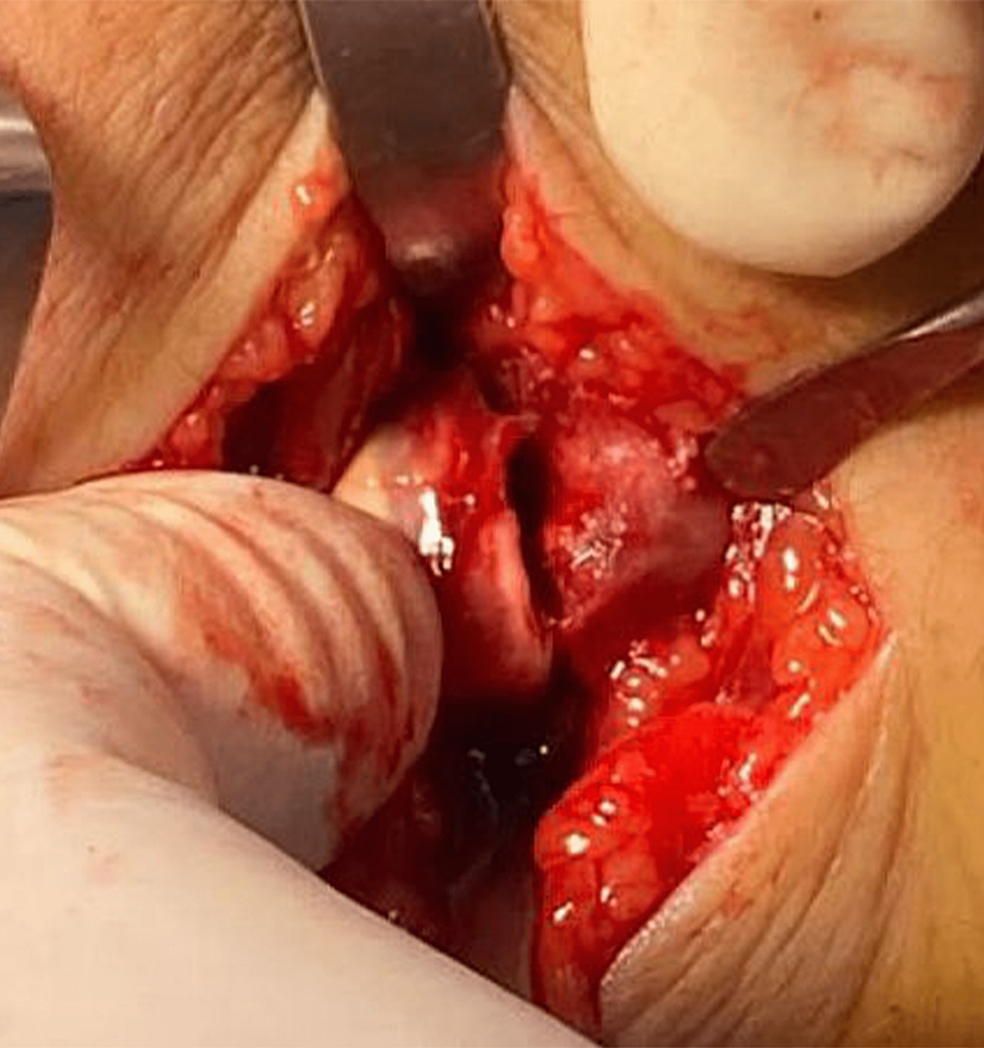
Open reduction of distal ulnar physeal fracture.

**Figure 10:**
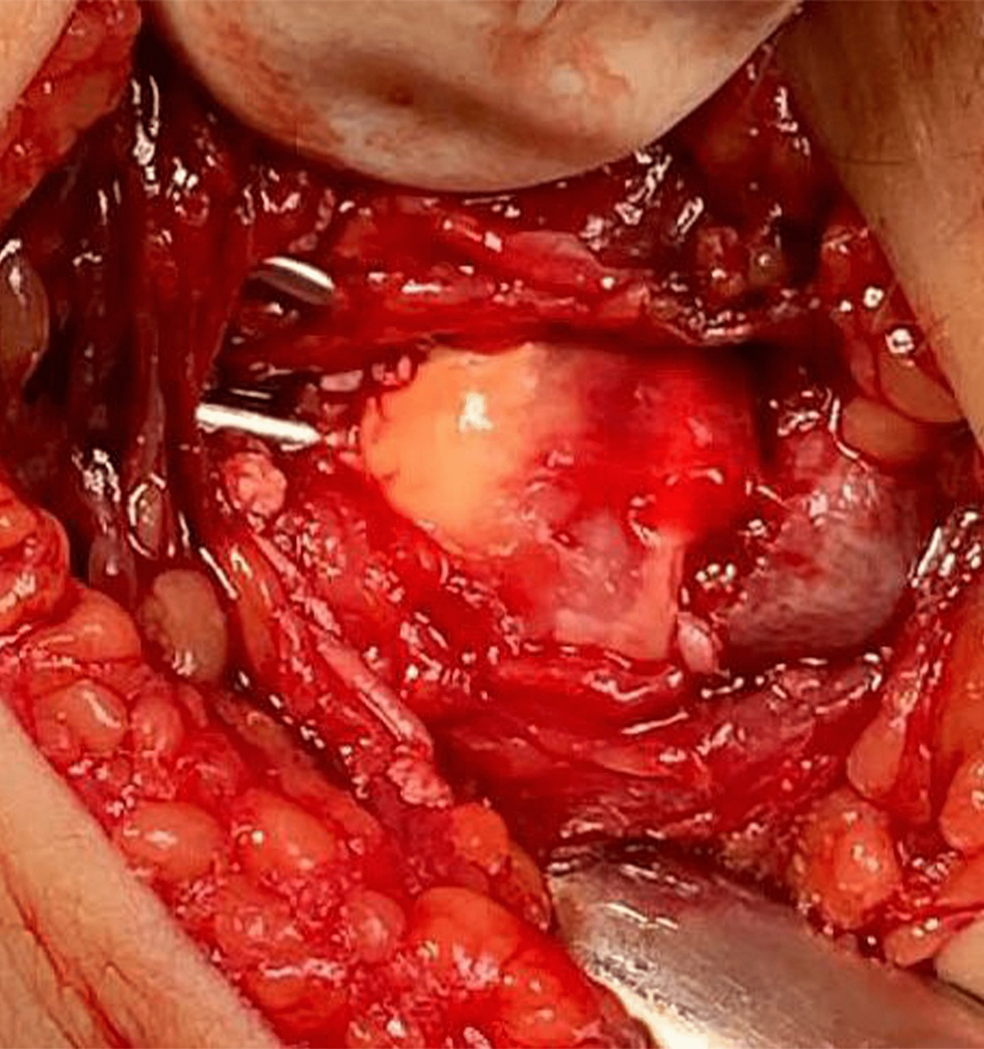
Fixation of distal ulnar physeal fracture by two K-wires.

On the day of surgery, the patient was assessed preoperatively for general anesthesia. Preoperative antibiotics were administered with the induction of anesthesia. We placed him in a supine position and prepping and draping were done. For the radius fracture, we used a modified Henry approach with an incision 7 cm over the fracture site. The fracture was exposed and reduced, fixed by locking compression plate (LCP) 3.5 with seven holes and three bicortical screws proximal and three screws distal to the fracture. The ulna fracture was irreducible after fixation of the radius, so open reduction and removal of interposed tissues were necessary. An incision around 4 cm over the ulna styloid was made, the fracture was exposed, irrigated to clean the hematoma, and tissue was interposed without using a curette to preserve the growth plate. The reduction was achieved (Figure 9) and fixed by two crossed 1.5 mm smooth K-wires (Figure 10).

DRUJ assessment was done after fixation, and it was stable. Closure was performed in a routine manner, and a back slab was applied above the elbow.

**Figure 11:**
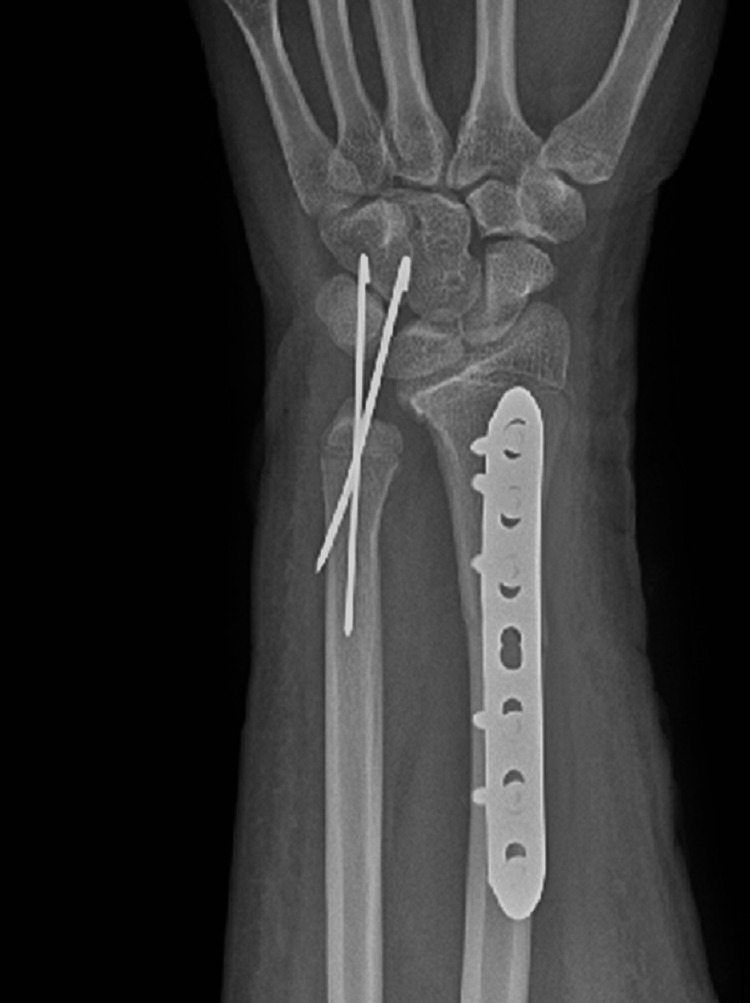
Postoperative PA wrist view. PA: posterior-anterior.

**Figure 12:**
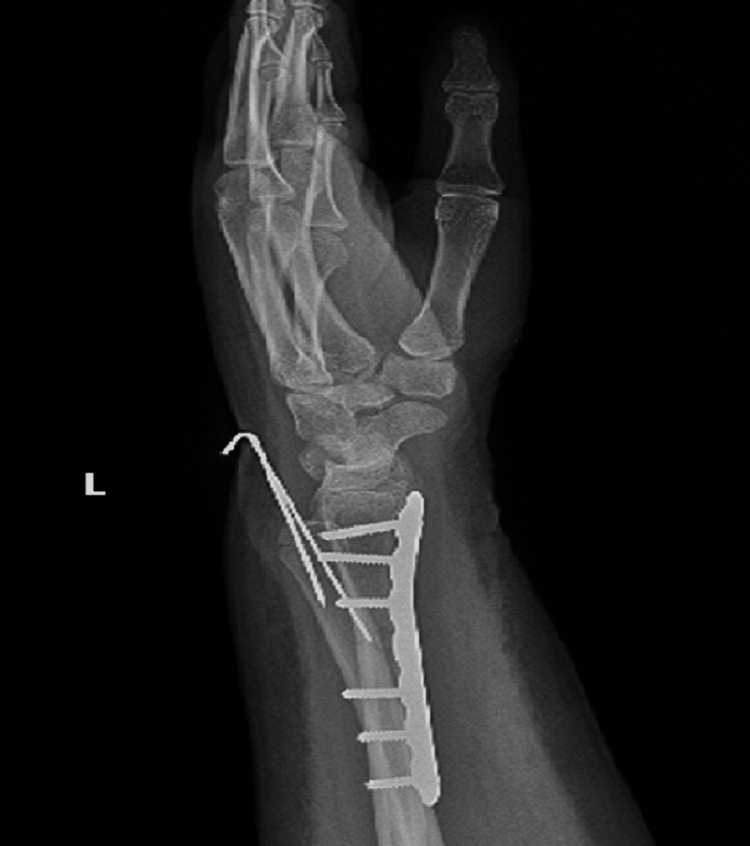
Postoperative lateral wrist view.

Postoperative X-rays showed both fractures were properly reduced and well fixed (Figures 11, 12). The lateral wrist view showed minimal volar apex bowing of the distal ulna shaft, which was comparable to the contralateral wrist view (Figure 6).

**Figure 13:**
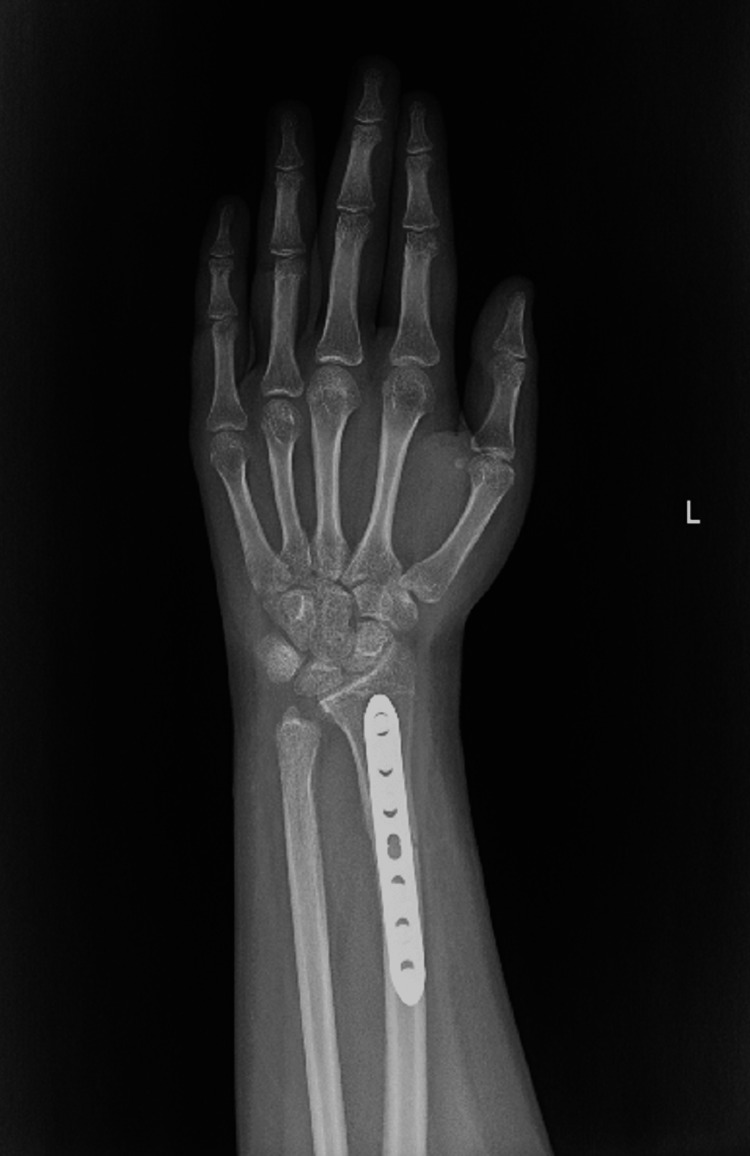
PA wrist view post K-wire removal. PA: posterior-anterior; K-wire: Kirschner wire.

**Figure 14:**
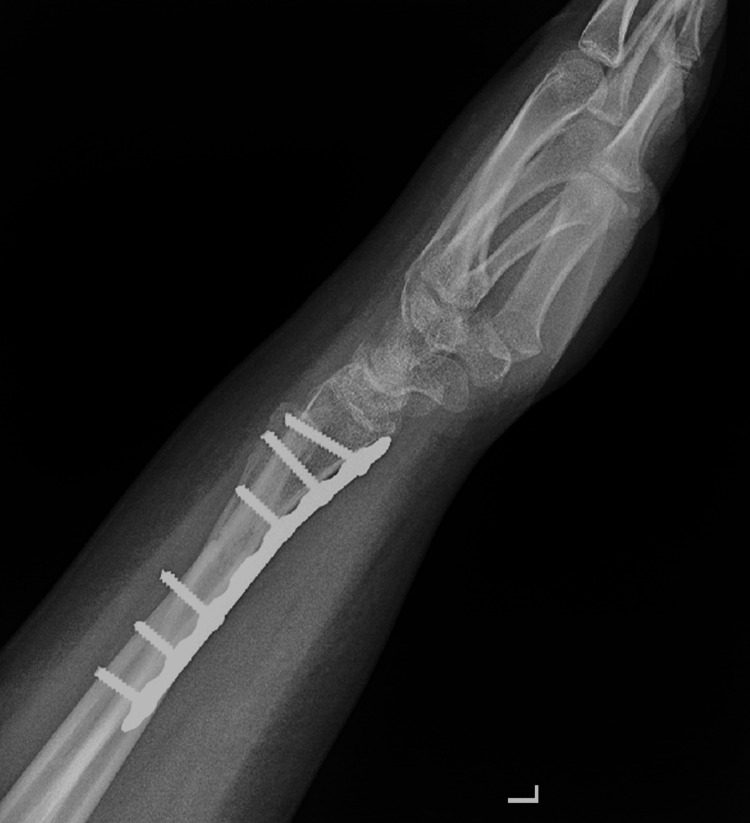
Lateral wrist view post K-wire removal. K-wire: Kirschner wire.

**Figure 15:**
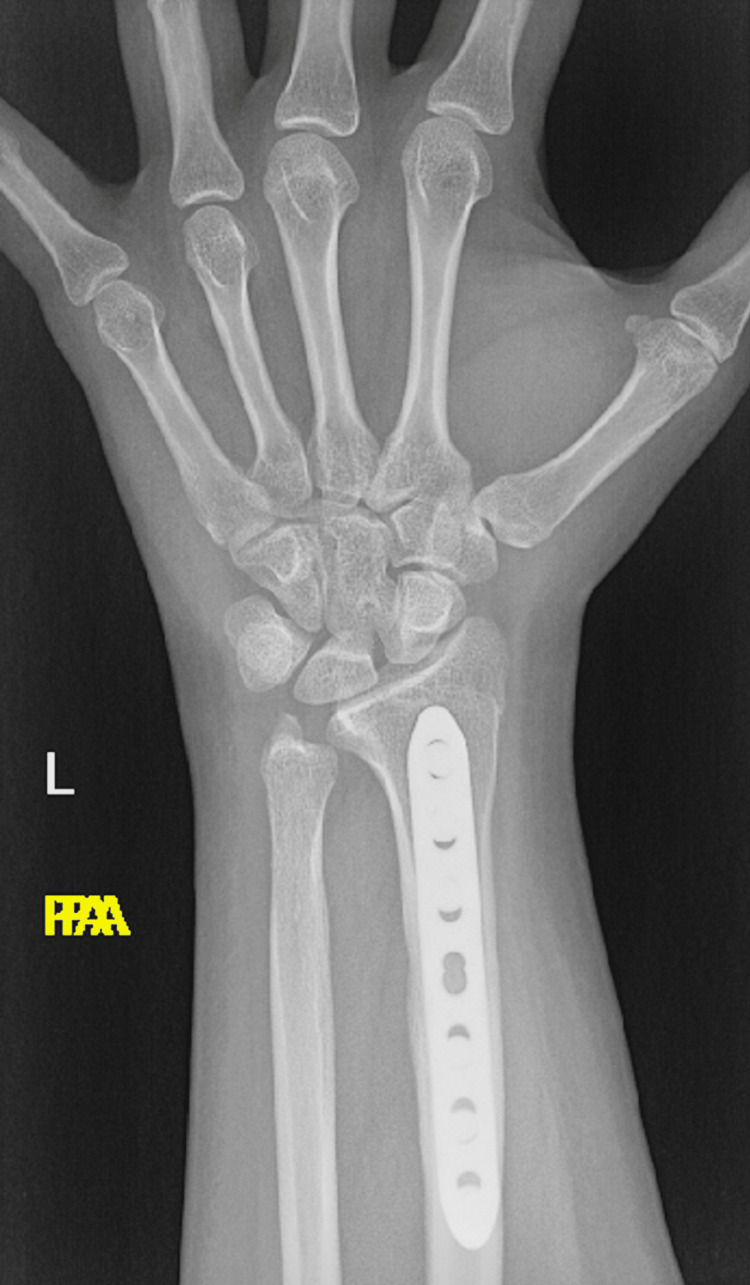
Six-month postoperative PA wrist view. PA: posterior-anterior.

**Figure 16:**
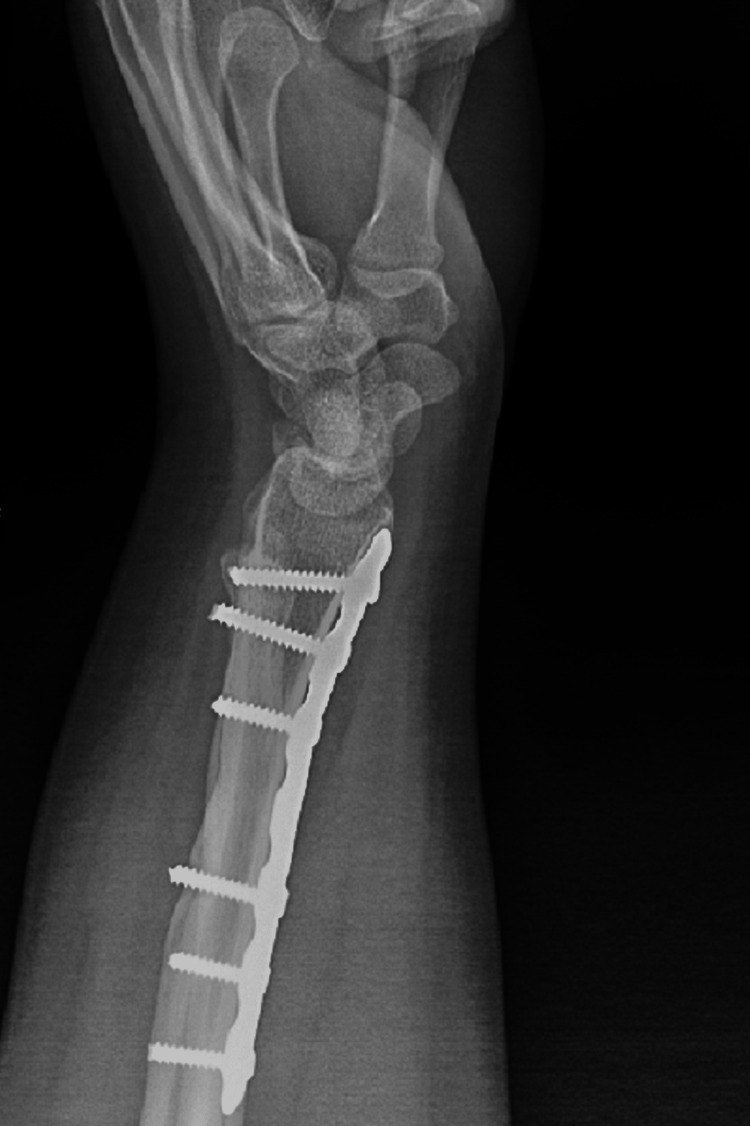
Six-month postoperative lateral wrist view.

The patient was seen postoperatively at two, four, and six weeks. At two weeks follow-up, the suture was removed and the splint changed to a below-elbow cast. At six weeks, the K-wires were removed (Figures 13, 14). Complete bone union was achieved, and a normal range of motion was apparent six months postoperatively (Figures 15, 16). The patient was able to perform daily and sports activities. At two-year follow-up, complications such as DRUJ instability or joint deformity have not occurred.

## Discussion

It is crucial to recognize Galeazzi-equivalent fractures when considering distal ulnar physeal injuries with a risk of growth arrest. Galeazzi-equivalent fractures have an intact DLSS; therefore, the stability of the DRUJ is maintained by reducing the distal ulnar fracture. Treatment with closed reduction and application of an above-elbow cast in cases are easily reduced with minimal initial displacement [4].

In some cases, there is a failure to reduce the ulnar epiphyseal plate injury, mainly because of the interposition of soft tissues such as the periosteum, extensor tendons, or the capsule [6]. With distal radius fracture reduction, most of the distal ulnar physeal fractures will be reduced to an acceptable alignment. If it is irreducible, open reduction and pinning should be performed [7].

Pronation type is a rare pattern. A case reported by Ashish Suthar and Ashish Kothari showed an acceptable close reduction for a radius fracture, which was fixed by crossed K-wires, whereas the ulnar epiphyseal fracture was irreducible by close reduction. Open reduction and fixation by two K-wires were performed [8].

In contrast to Ashish's case, a case of pronation pattern injury reported by Chae and Kwon had difficulties in a close reduction for a radius fracture due to soft tissue interposition; however, a close reduction for the ulnar epiphyseal fracture was achieved and fixed using a percutaneous pinning technique [9]. In our case, the close reduction was unsatisfying, with an unstable fracture pattern. Open reduction and internal fixation of the distal radius fracture were achieved, as the stabilized anatomical reduction of the radius fracture facilitated distal ulna physeal fracture reduction [7]. Considering the patient was older than most children who have the same injury pattern, he had limited potential for bone remodeling, with less than two years of growth remaining [9,10]. However, the ulnar epiphyseal fracture was irreducible by close reduction after stabilizing the radius fracture. Open reduction and fixation by K-wires were performed. During open reduction of the ulnar epiphyseal plate, it is important to prevent more damage to the growth plate and avoid using a bone curette to clean the fracture site. We only washed the fracture site to clean the hematoma. However, DRUJ remains intact in Galeazzi-equivalent fractures. It is preferred to assess DRUJ stability after anatomical reduction of the radius and ulna fractures. If it is still unstable, additional radioulnar pinning is required. Immobilization with an above-elbow splint is necessary for extra stability. We changed it to a below-elbow cast two weeks postoperatively to avoid elbow joint stiffness with prolonged immobilization.

The risk of growth disturbance of the ulna following epiphyseal injuries has been reported in 55% of Galeazzi-equivalent fracture cases [5]. Two out of 13 cases had growth arrest without functional disability [11]. The anatomical reduction of the distal ulna is essential to reduce the risk of growth plate disturbance. Eight out of 10 patients aged 11-16 years showed negative ulnar variance during average follow-up periods of 18 months [12].

At six months of follow-up, our case had a full range of motion and returned to normal daily and sports activities. At two years of follow-up, complications such as DRUJ instability or joint deformity have not occurred. Regular serial follow-up sessions at six to 12 months after trauma and further follow-up over a year to assess growth arrest and the occurrence of other complications, such as accompanying deformity, are necessary.

## Conclusions

Galeazzi-equivalent fractures are complex injuries that require careful diagnosis and treatment. It is important to analyze and define these fractures accurately to avoid complications such as growth arrest, which has been reported in such injuries. In some cases, it may be necessary to obtain radiographs of the opposite uninvolved extremity to accurately identify the injury. Open reduction may also be required for Galeazzi-equivalent lesions with malalignment or older patients who have less potential for sufficient bone remodeling. However, open reduction must be performed with care to avoid further injury to the physis. Regular serial follow-up sessions are required to assess growth arrest and the occurrence of other complications.
